# Effect of Agroindustrial Waste Substrate Fermented with Lactic Acid Bacteria and Yeast on Changes in the Gut Microbiota of Guinea Pigs

**DOI:** 10.3390/microorganisms12010133

**Published:** 2024-01-10

**Authors:** José Miranda-Yuquilema, Juan Taboada, Verónica Once, Marco Coyago, Wilfrido Briñez

**Affiliations:** 1 Animal Production and Industrialization Research Unit, Engineering Faculty, Universidad Nacional de Chimborazo, Riobamba 060150, Ecuador; 2 Faculty of Agricultural Sciences, Universidad de Cuenca, Cuenca 010205, Ecuador; juan.taboada@ucuenca.edu.ec (J.T.); veronica.oncec@ucuenca.edu.ec (V.O.); livemarco88@gmail.com (M.C.); 3 Faculty of Veterinary Sciences, Universidad del Zulia, Maracaibo 4001, Venezuela; wilfido.brinez@luz.edu.ve

**Keywords:** probiotic, production, intestinal health, performance, intestinal flora

## Abstract

The aim of the study was to evaluate the impact of probiotics obtained from an agroindustrial waste substrate fermented with lactic acid bacteria and/or yeasts on the health and changes in the microbiota of the digestive tract of guinea pigs. Eighty male guinea pigs, Kuri breed, 30 days old and 250 g live weight, were randomly selected and divided into four groups of 20 animals each: T0, control; T1, *Lactobacillus acidophilus* and *L. bulgaricus*; T2, *Saccharomyces cerevisiae* and *Kluyveromyces fragilis;* and T3. *L. acidophilus*, *L. bulgariccus, S. cerevisiae* and *K. fragilis*. T1, T2 and T3 contained molasses-vinasse substrate in their base, the dose administered was 1.00 mL/animal orally every 3 days. The indicators evaluated were weight gain, occurrence of diarrhea and mortality, macroscopic lesions in the digestive tract organs and changes in the microbiota of the stomach, caecum, small and large intestine. Treatments T1, T2 and T3 improved weight gain (*p* < 0.05) and reduced the presence of guinea pigs with diarrhea (*p* < 0.05) and there was no mortality; animals in the control group presented a greater amount (*p* < 0.05) of macroscopic lesions in the digestive tract organs; in the T1, T2 and T3 groups there was an improvement in the natural microbiota. It is concluded that the inclusion of a microbial additive in young guinea pigs improves intestinal health and consequently improves weight gain, reduces diarrhea and deaths and normalizes the natural microbiota of the gastrointestinal tract.

## 1. Introduction

In the Andean countries over the last decade, the consumption of nonconventional animal proteins such as guinea pig (*Cavia porcellus*) meat has been increasing, which has led to a significant increase in the production of this species in the rural sector in Ecuador [1]. In Ecuador, in the last two decades, per capita consumption of guinea pig meat has increased from 0.7 to 2.5 kg/year/person. The increase in demand for this meat has been due to its nutritional value [2,3]. However, the feed efficiency of guinea pigs reared in Ecuador is still not ideal, because producers in this sector have not yet implemented feeding strategies to improve animal performance [1,3].

In most developing countries, the use of additives such as antibiotic growth promoters still persist in animal production, with the aim of improving animal health and increasing the profitability of animal production [2,4]; however, the inappropriate use of these products has led to antimicrobial resistance, attributed to antibiotic residuals in the carcass, which creates a public health problem [2,5].

Probiotics are composed of lactic and non-lactic (fermenting) bacteria, yeasts and fungi. These microbial additives have beneficial properties for animal health and their main action on the host is to improve weight gain, increase feed conversion and modify the intestinal flora [6,7]. They also help to make the animal more resistant to disease and reduce the use of antibiotic growth promoters, so it is important to include them in the diet of monogastric or ruminant animals [8,9].

The use of microbial bioactive compounds such as probiotics, prebiotics and synbiotics emerges as a viable alternative to antibiotic growth promoters [10], with the premise of maintaining meat safety, improving animal welfare, the development of the gastrointestinal tract and the immune system [11] and improving carcass yield without leaving residues in the carcass [12]. Recent studies provide encouraging data on the inclusion of diets with beneficial microorganisms having the ability to balance the microbiota in the different segments of the gastrointestinal tract, which has attracted much attention from researchers [2]. However, scientific information on the positive effect of beneficial microorganisms and the mechanism of action is still insufficient [9,13].

Previous studies on the use of agroindustrial waste (molasses-vinasse) substrates fermented with lactic acid bacteria and yeasts have shown significant (*p* < 0.05) increases in weight gain in pre-weaned and post-weaned piglets, chickens and cattle [7,13,14]. It has also been shown to improve health by reducing diarrhea disorders and deaths. However, there are still no scientific data showing a positive action on productive efficiency, health and the ability to balance the natural microbiota in the different segments of the organs of the gastrointestinal tract in growing guinea pigs [15,16].

The inclusion of microbial bioadditive compounds obtained from agroindustrial waste fermented with lactic acid bacteria and yeasts in the diet of growing and fattening guinea pigs will lead to a reduction in diarrhea and deaths caused by pathogens, an increase in weight gain, a reduction in macroscopic lesions of the digestive tract organs and a modification of the natural microbiota. Fortunately, thus far there are no reports of negative effects of probiotics in animals; therefore, the use of microbial bioadditive compounds in feed is encouraging for the future, and they are postulated as a good alternative for the replacement of growth-promoting antibiotics [2,7,17,18].

While it is true that studies related to the composition and possible changes in the gut microbiota of animals when probiotics are used, by means of biochemical tests, nowadays offer an approximation to their distribution and abundance, until recently it was almost completely unknown what changes can be generated in the natural microbiota of the host’s digestive tract [5,9,12]. 

However, the microbial modifications in the different segments (stomach, small intestine, cecum and colon) of the digestive tract of guinea pigs when they are included in the diet with agroindustrial waste substrate fermented with lactic acid bacteria and yeasts are still almost completely unknown. Therefore, the aim of the study was to evaluate the impact of probiotics obtained from agroindustrial waste substrate fermented with lactic acid bacteria and/or yeasts on the health and changes in the microbiota of the digestive tract of guinea pigs.

## 2. Materials and Methods

### 2.1. Animal Bioethics and Place to Study

All experimental procedures applied in this study were reviewed and approved by the Commission of Scientific Degrees by Agreement N° 189/13-14. Faculty of Agricultural Sciences, University of Zulia. 

The procedures related to the handling, management and health care of live guinea pigs complied with the standards applicable to laboratory animals used for scientific purposes and were applied in accordance with the minimum standards for the protection of animals described in Council Directive 2008/120/EC on the minimum standards for the protection of pigs (Council of the European Union, 2008) [19].

The animal experimental study was carried out at the “Irquis” farm, located at Km 20 via Salado-Lentag, Cuenca, belonging to the Universidad de Cuenca.

### 2.2. Selection, Strain Activation and Biomass Production

The strains used were: *Kluyveromyces fragilis* 6.4 × 10^8^ CFU/mL from the Strain Bank of the Universidad Central “Marta Abreu” de Las Villas, Cuba, and three strains American Type Cultures Collection (ATCC) (Global Bioresourse Center, EEUU) of the genus: *Lactobacillus acidophillus* (ATCC 4356) 5.6 × 10^7^ CFU/mL, *Lactobacillus bulgaricus* (ATCC 11842) 5.5 × 10^8^ CFU/mL and *Saccharomyces cerevisiae* (ATCC 18824) 6.2 × 10^7^ CFU/mL. The strains, in lyophilized format, were individually activated in 120 mL of tryptone soy broth (BD Difco™, Trypticase, Texas, EEUU) at 37 °C for bacteria and 30 °C for yeast, in an oven with an orbital shaker (Inkubationshaube TH 15, GmbH, Bodelshausen, Germany) at 60 rpm for 6 h. They were subsequently grown on plates with Man, Rogosa and Sharpe (MRS) agar (BD Difco™, MRS Agar, Texas, EEUU) and Nutrient (BD Difco™, Nutrient agar, Texas, EEUU) culture medium for *L. acidophilus* and *L. bulgaricus*, respectively. Sabouraud Agar (BD BBL™, Sabouraud Dextrose Agar, Texas, EEUU) was used for yeasts. Lactobacilli were cultured under anaerobic conditions using the jar (BBL^®^, GasPak Plus™, Pleasant Prairie, EEUU). Once the strains were activated, a microbial biomass was obtained using a standard medium (Nutrient agar and MRS agar) for strain growth. The culture was composed of 5 mg (Analytical Balance Radwag, AS 220.X2 PLUS, Radom, Poland) of each of the strains *L. acidophilus*, *L. bulgaricuc*, *S. cerevisiae* and *K. fragilis*. Subsequently, the microorganisms were mixed in 250 mL of inoculum (skimmed milk) at 30 ± 2 °C and incubated at 37 °C for 24 h. Finally, an initial plate count was performed to verify the viability of the strains [20].

### 2.3. Obtaining Substrates from Agroindustrial Waste Fermented with Bacteria and Yeasts

The molasses came from the Ingenio Azucarero Valdez (Milagro, Ecuador), while the grape vinasse was obtained from the Baldoré wine company (Patate, Ecuador). The microbial bioadditive was obtained at the following concentrations 33% (*w*/*w*) molasses, 54.5% (*w*/*w*) vinasse and 12.5% (*w*/*w*) microbial inoculum previously obtained, as mentioned in Table 1. 

All treatments were independently homogenized at 150 rpm with a magnetic stirrer (Magnetic Stirrer, model HSC-19T, JOANLAB^®^, Huzhou, China) at 28 °C for 10 min. The chemical composition of vinasse was 20, 16, 10.85 and 1.85% dry matter (DM), crude protein (CP), true protein (TP) and ash, respectively; while molasses contained 78.65, 2.8, 0.8 and 1.1% DM, CP, TP and ash, respectively; the °Brix of molasses was 84 and that of vinasse 7.32. Proximate chemical analysis was performed according to the methodology described by AOAC International [21].

The 5 L of microbial substrates (T1, T2 and T3) used in the study was stored in 6 L dark glass bottles in a cool and dry place at a temperature of 12 ± 2 °C, for 100 days.

### 2.4. Design and Dosage of Probiotics Used in the Study

Using a completely randomized design, four treatments were carried out, four replicates per treatment, each replicate consisting of 5 animals. The treatments evaluated are shown in Table 1.

### 2.5. Animals, Feeding, Farm Layout and Management System

The study used 80 male guinea pigs (*C. porcellus*), Kuri breed, 30 ± 5 days old and 250 ± 30 g live weight.

The feed (basal diet) offered to the study animals was a mixture of 20% alfalfa (*Medicago sativa*), 25% maralfalfa (*Pennisetum* spp.), 30% king grass (*Pennisetum purpureun* x *P. typhoides*), 24.97% fattening guinea pig feed and 0.03% vitamin C. The diet was designed for three phases: phase I from 30 to 60, phase II from 61 to 90 and phase III from 91 to 120 days of age (Table 2).

Feed was provided in two equal rations per day (07:00 a.m. and 04:00 p.m.), as recommended in a previous study by Miranda [22], in accordance with the recommendations described in the National Research Council (NRC) [23] that meet the minimum requirements established for guinea pigs. In addition, 50 mL of water was offered daily in automatic waterers (Niple–Cuy, Latacunga, Ecuador).

The biosecurity measures on the farm were conditioned prior to the reception of the guinea pigs, as recommended by Vivas [24], which allowed animal health control of the animals during the study. Site disinfection was performed at a dose of 3 mL/L with C_5_H_8_O_2_ (Glutaraldehyde), quaternary ammonium and isopropyl alcohol (Viroguard^®^6, Agrovet Market Animal Health, Lima, Perú), as recommended by the manufacturer.

Guinea pigs were housed in group cages of 1.50 × 1.00 m^2^, with five animals per cage. The temperature of the house was maintained at 14 ± 2 °C. The cages for each treatment were placed 1.50 m apart on both sides of the aisle to avoid self-inoculation. All animals subjected in the study received the relevant veterinary care according to the guinea pig (*C. porcellus*) husbandry manual [24].

### 2.6. Obtaining and Administering Microbial Bioactive Compounds to Guinea Pigs

The administration of the microbial bioactive compounds under study was carried out according to the dose and group indicated in Table 1, the first dose was in single doses orally by syringe and from the second dose every 3 days, microbial bioactive compounds were inoculated in 25 g of balanced feed; this was administered in the morning hours according to the dose and group assigned (Table 1) and the animals in the control group received 1 mL of distilled water.

### 2.7. Productive Indicator, Clinical Evaluation, Culling and Visceration of Guinea Pigs 

The guinea pigs under study were weighed on a digital scale (KAMRY, JCM, Bogota, Colombia) of 5.00 kg capacity with an error of ±5 g, at baseline (30 days of age) and at 30, 60 and 90 days of study; with this information, weight gain (WG) was calculated.

All guinea pigs under study were monitored daily for macroscopic lesions, behavioral changes and health status. The presence of diarrhea and deaths was recorded daily on an individual basis; with this information, the percentage of diarrhea and mortality was calculated as described by Thrusfield [25] and Miranda [7].

On Day 90 of the study, 24 guinea pigs (n = 6 per treatment) were randomly selected to be sacrificed with prior fasting for 12:00 h and followed by stunning [26] were sacrificed by the atlanto-occipital joint dislocation method described by Sánchez [27] and Mínguez [28], previously established in the Mexican Official Standard NOM-033-ZOO-1995, Humane Slaughter of Domestic and Wild Animals (Humane Slaughter Association, 2015) [29].

After slaughter, the removal of hair and manipulation of the guinea pig carcasses was performed following the methodology described by Sánchez [27] and Mínguez [28]. Evisceration was performed by laparotomy; the following organs were carefully removed from the mesentery for the study: lungs, spleen, thymus, kidneys, stomach and small and large intestine. The latter three organs were washed with distilled water after the removal of the cecal contents. The previously obtained digestive tract organs were weighed on a calibrated digital electric balance (KAMRY, JCM, Bogota, Colombia) with a capacity of 5.00 ± 0.1 kg.

### 2.8. Gross Pathological Examination of the Organs of the Gastrointestinal Tract

The evaluation of macroscopic lesions in guinea pigs was performed by measuring the following parameters: degree of thickness of the intestinal wall, presence of circulatory disorders in the mucosa (edema, congestion, hemorrhages) and consistency of the intestinal contents (watery, mucous, foamy), according to the methodology described by Canal [30]. The pH values of the intestinal contents were measured using a digital pH meter (Hanna^®^, Pandova, Italy).

### 2.9. Collection of Intestinal Mucosa and Culturing on Selective Microbial Growth Media 

To obtain the mucus samples, a 2.00 cm^2^ longitudinal incision of the stomach, small intestine, cecum and colon was made and washed with sterile distilled water and saline phosphate buffer (BFS) according to the methodology used by Cueto [31]. 

The previously obtained fragments were deeply scraped with the help of a 75 mm spatula until 2.00 mL of intestinal mucus was obtained; these samples were deposited in 15 mL Falcon plastic tubes (Sterile falcon, Greiner, Germany) with sterile screw cap and 5.00 mL of BFS was added according to the methodology described by Kandler and Weiss [32]. Finally, they were centrifuged (digital centrifuge, Yingtai, China) at 4582× *g* at 8 °C for 10 min and the supernatant was removed; this procedure was performed three times.

A total of 1.00 mL of the previously obtained sample was added to a 150 mL Erlenmeyer flask containing 50 mL of nutrient broth and MRS, separately and independently of each organelle. They were then incubated at 37 °C for 6 h in an incubator with an orbital shaker (Inkubationshaube TH 15, GmbH, Bodelshausen, Germany) at 15 rpm [32]. After this time, 5.00 mL of each culture was taken and homogenized with physiological saline at a ratio of 1/10 (*v*/*v*), followed by serial dilutions of 1/10, (*v*/*v*) to the 0.5 scale of the MacFarland scheme.

From the previously obtained dilutions, the cultivation was carried out by the striation depletion method in Petri dishes containing MRS agar, M17, Sabouraud Dextrose, MacConkey, sterile selective media and a general Nutrient culture medium, separately. Petri dishes with Nutrient and MacConkey agar were then incubated at 37 °C for 48 h and Sabouraud Dextrose agar at 30 °C for 72 h, while MRS and M17 were incubated under anaerobic conditions in a flask (BBL^®^, GasPak Plus™, Pleasant Prairie, EEUU) with 5% CO_2_ for 48 h. After this time, typical colony growth was checked. Finally, Gram staining was performed and observed with a binocular optical microscope (BA310 MOTIC, Motic^®^ Hong Kong, China) to differentiate morphotintorial characteristics [32].

### 2.10. Identification with API System 50 CHL, 20NE and ID 32 C

The commercial systems API 50 CHL, API 20NE and ID 32 C were used according to the manufacturer’s instructions. Incubation for API 50 CHL and 20NE was 24 to 48 h under anaerobic conditions for the former and aerobic conditions for the latter at 37 °C; while for ID 32 C, it was 72 to 96 h at 30 °C. After reading the reactions produced, spontaneous or revealed by the addition of reagents, the data were manually recorded for subsequent incorporation into the APIWEB^TM^ software (https://apiweb.biomerieux.com/ accessed on 15 September 2022). The results obtained were reported according to the criteria established by the manufacturer, considering a result as valid when the identification percentage was at least 90%. The identification results were classified into the following quality categories: excellent (***), very good (**) and good (*).

### 2.11. Statistical Analysis

Experimental data were processed with the statistical package Statgraphics plus ver. XV. II for Windows. Experimental variables such as weight gain, relative weight of digestive tract organs and microbial load (CFU·mL^−1^) obtained in the culture media were subjected to a simple rank analysis of variance (ANOVA) according to a completely randomized design [33]. When the *p*-value was *p* < 0.05, a Duncan [34] comparison test was applied to discriminate differences between treatments. 

For the variables occurrence of diarrhea and percentage mortality, a multiple comparison analysis of proportions was performed in the statistical package SAS version 17.

## 3. Results

**Figure 1:**
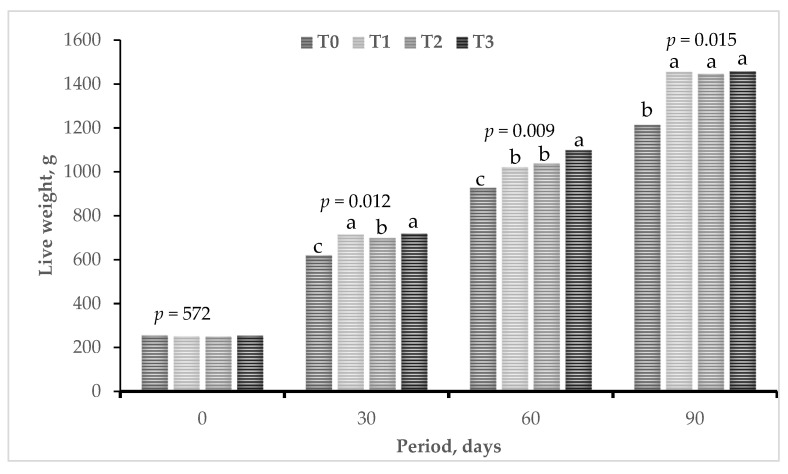
Weight gain of guinea pigs in the evaluations carried out at the beginning, and at 30, 60 and 90 days of the study. ^a,b,c^ different letters in the bars differ at *p* < 0.05 [34]. T0, control. T1, *L. acidophilus* and *L. bulgaricus*. T2, *S. cerevisiae* and *K. fragilis*. T3. *L. acidophilus*, *L. bulgaricus*, *S. cerevisiae* and *K. fragilis*.

Figure 1 summaries the responses in weight gain in guinea pigs. At initial weighing, guinea pigs showed no significant differences (*p* = 0.572) between treatments. 

However, in the evaluation carried out at 30 days of study, the animals that consumed the agroindustrial substrate fermented with lactic (fermenting) bacteria and yeast (T1, T2 and T3) improved their weight gain, but the animals in the treatments T1 and T3 gained more weight (*p* < 0.012) compared to the other treatments. The treatment T3 also performed better in weight gain, followed by T1 and T2 at 60 days of study (*p* < 0.009).

In the evaluation carried out at the end of the study (90 days of study), the guinea pigs of the control treatment presented a lower weight (*p* < 0.015) compared to the animals that consumed agroindustrial substrate fermented with lactic bacteria and yeast (T1, T2 and T3). This shows that probiotic microorganisms have a positive action on the utilization of the diet offered.

The animals that consumed diets with microbial additives presented less (*p* < 0.05) incidence of diarrhea, and of these the treatments T1 and T2 presented less (*p* < 0.015 and *p* < 0.014) occurrence of diarrhea, in the evaluation carried out at 30 and 60 days, respectively. However, the species of pathogenic micro-organisms causing diarrhea at this stage were not identified. 

In terms of deaths, the animals in the control group (T0) had a higher number of dead guinea pigs (n = 4) due to diarrheal disorders at 30 and 60 days of the study. In the same period, there were two dead animals in the T1 treatment. In T2 and T3, there were no dead animals; but there were no guinea pig deaths at 90 days of the experiment (see Table 3).

Table 4 shows the general weight status of the digestive tract organs of guinea pigs at 120 days of age. The weight of the small intestine with cecal contents of the animals in the T1 and T3 treatments was higher (*p* = 0.012), compared to the T2 and control group. The liver weights were lower (*p* = 0.012) in animals not treated with the microbial bioactive compounds (T1, T2 and T3). The lung weights of guinea pigs consuming the T2 and T3 treatments were higher (*p* = 0.008) compared to T0 and T1. The kidney weights of the treatment T3 were higher than those of T2, T1 and T0. However, in the other organs evaluated there were no significant differences (*p* > 0.05).

Table 5 shows the results of macroscopic lesions of the digestive tract organs in 120-day-old guinea pigs. The control treatment (T0) showed a higher number of guinea pigs with lesions at the intestinal level. However, guinea pigs that consumed microbial bioactive compounds fermented with lactic acid bacteria and yeasts (T1, T2 and T3) did not show well-defined macroscopic lesions to be considered physiological alterations at the level of the digestive tract organs.

The stomach pH values in the control group animals were higher than 2.9, while in the treated animals (T1, T2 and T3) they were lower than 1.8. The pH of the small intestine in animals treated with microbial bioactive compounds was lower than 4.8, in contrast to the control group (pH > 5). In the cecum and colon, pH values were higher than 6 in animals of the control group, compared to animals of the T1, T2 and T3 groups (pH < 5.8), as shown in Table 5.

Macroscopic changes in the stomach, small intestine, colon and cecum showed significant changes in relation to the thickness of the intestinal wall in the animals of the control group (T0) compared to guinea pigs consuming the agroindustrial waste substrate fermented with lactic acid bacteria and yeast. A similar situation occurred with circulatory disorders at the level of the intestinal mucosa and contents in the T0 treatment, as can be seen in Table 5.

Table 6 presents the microbial load cultured on *Lactobacillus* spp., *Kluyveromyces* spp. and *Saccharomyces* spp. and *Enterobacteriaceae*. In stomach contents samples (post-mortem) cultured on *Lactobacillus* spp., *Kluyveromyces* spp. and *Saccharomyces* spp., there was no significant growth (*p* > 0.05) between treatments. However, on *Enterobacteriaceae*, higher microbial growth (*p* = 0.003) was observed in samples from the control treatment (T0) compared to the other treatments (T1, T2 and T3).

Samples of small intestine, colon and cecum scrapings from animals consuming diets containing the agroindustrial waste substrate fermented with lactic acid bacteria and yeast (T1, T2 and T3) showed increased microbial growth (*p* < 0.05) when cultured on Petri dishes containing *Lactobacillus* spp., *Kluyveromyces* spp. and *Saccharomyces* spp., compared to guinea pigs from the control treatment. In contrast, for *Enterobacteriaceae*, higher microbial growth was observed in the control treatment samples compared to the T1, T2 and T3 treatments (see Table 6).

Table 7 reports the main microorganisms detected in the different organs of the digestive tract in guinea pigs at 120 days of age. In the samples from animals that consumed a diet containing the agroindustrial waste substrate fermented with lactic acid bacteria and yeasts (T1, T2 and T2), a higher presence of microorganisms with numerical profiles corresponding to *L. acidophilus* (T1 and T3), *L. bulgaricus* (T1 and T3), *Saccharomyces* spp. (T2 and T3) and *K. fragilis* (T2 and T3) was observed compared to animals in the control group. 

In samples from control treatment animals, a higher presence of numerical profiles corresponding to pathogens such as *E. coli* was observed.

In the small intestine scraping samples, a higher presence of numerical profiles of microorganisms known as probiotics was observed mainly in the T1, T2 and T3 treatments, compared to samples from animals in the control group, as shown in Table 7.

## 4. Discussion

### 4.1. Productive Behaviour

In guinea pigs consuming bioactive microbial compounds containing lactic acid bacteria and yeasts in the diet, a significant increase in weight gain was observed (Figure 1), which may have been due to the action of the probiotic microorganisms in the digestive tract, where they possibly aided in the regeneration of atrophied or damaged microvilli in the small intestine and consequently improved nutrient absorption. Similar results were also obtained by Puente [35] when using probiotics as an alternative to antibiotic growth promoters in animal feed. However, Aristides [12] did not observe any significant variation in weight gain when commercial probiotics were included in broiler feeds. However, pigs fed probiotics containing different *Lactobacillus* species showed an improvement in daily weight gain and, at the same time, a reduction in the incidence of diarrhea compared to the control group [9,15,36].

The increased weight gain in animals consuming agroindustrial waste substrate fermented with lactic acid bacteria and yeasts could be due to the presence of microorganisms with probiotic action in the small intestine, where they acted positively in the small intestine, resulting in weight gain in guinea pigs [1,37]. In addition, there are reports of increased enzyme activity, mainly amylase, sucrase and lactase, when probiotics obtained from lactic acid bacteria (*Lactobacillus*) were administered in piglet and guinea pigs diets, which could improve digestion and absorption of nutrients available in the intestinal lumen [5,38,39].

Probiotics reduce the symptoms of lactose maldigestion [40]. It is known that probiotics can reduce the symptoms of malabsorption in animals [9,11,14,39]. This effect is due, on the one hand, to the fact that the probiotic microorganisms (bacteria and yeasts) contained in these products have mechanisms of action such as participation in immune processes [17,41]. 

Probiotics derived from *Lactobacillus* strains are known to release protective agents such as enzymes and bacteriocins, and enzymes are able to modify toxin receptors and thus block toxin-mediated signaling pathways, while bacteriocins inhibit the growth of other pathogenic species [5,16]. In addition, they produce lactic acid which lowers pH at the intestinal level, (an effect also seen in this study, Table 5), hence promoting the enzymatic activity of proteases, lipases and amylases [6,18], thereby improving digestion and nutrient absorption [10,16].

Cornejo [26] and Miranda [7] also reported positive effects on weight gain with the use of probiotics obtained from mixtures of lactic acid bacteria and/or yeasts in the diet of rabbits and pigs, mentioning that the increase in weight gain in the animals is due to the increase in enzyme activity caused by the probiotics. Therefore, it could be suspected that the use of agroindustrial waste substrate fermented with lactic acid bacteria and yeasts improves nutrient absorption and leads to increased weight gain in guinea pigs [8,41,42].

### 4.2. Health and Diarrhea

In the present study, guinea pigs consuming microbial bioactive compounds containing lactic acid bacteria and yeasts improved macroscopically, which at the same time reducing the macroscopic lesions in the gastrointestinal tract wall of the stomach, small intestine, colon and cecum relative to animals in the control group (Table 5). These results are consistent with the findings of Canal [30] describing macroscopic changes in the intestinal wall (mainly jejunum and ileum) in 1–7-day-old piglets infected with the *E. coli* strain, while others report to have observed a close relationship between pathogenic bacterial serogroups and hemolysin production.

In addition, the occurrence of diarrhea caused by enterobacteria was reduced in animals consuming the microbial bioactive compounds containing lactic acid bacteria and yeasts (Table 3 and Table 6). The mentioned effect in guinea pigs could be due to the increased presence of probiotic microorganisms in the digestive tract (Table 6). In relation to the above, it is known that the use of lactic acid bacteria (*Lactobacillus*) and yeasts (*Saccharomyces* and *Kluyveromyces*) are able to reduce diarrheal disorders caused by pathogens in piglets and chickens [14]. Other studies report similar results to those obtained in the present study when multi-strain probiotics are included in the animals’ diet [12,15].

According to other reports, the use of mixed cultures of lactic acid bacteria and yeasts, usually *Lactobacillus* alone or in combination with *Bifidobacteria*, *Enterococci* or *Saccharomyces* included in animal diets, reduces the risk of diarrhea associated with *E. coli*, *Salmonella* spp., among others [15,30]. While it is true that there is heterogeneity of results in terms of reducing diarrheal disorders in animals, there is insufficient evidence to say whether the effect varies systematically between the populations of microorganisms introduced in the probiotics used [8,16,30].

Similarly, controlled studies in which probiotics containing *Lactobacillus* spp., *L. bulgaricus* and *Streptococcus thermophilus* were administered to piglets and chickens reduced the incidence of *Clostridium* diarrhea [7,14]. According to the literature, the increase in pathogens in the digestive tract accumulates toxins in the epithelial mucosa; and the presence of microorganisms with probiotic action protects the epithelium through a cytoprotective effect and has the ability to increase mucin expression by cells in the ileum and colon; while the small intestinal epithelium synthesizes mucins to form a physical mucus barrier, which is a very effective mechanism in the fight against pathogenic bacteria [12,17,31].

Therefore, the administration of microbial bioactive compounds containing lactic acid bacteria and yeasts to guinea pigs is effective in preventing diarrhea associated with pathogenic bacteria [35]. In the case of the present study, different substrates were used. Thus, the inclusion of agroindustrial substrates fermented with multiple strains (*L. acidophilus*, *L. bulgaricus*, *S. cerevisiae* and *K. fragilis*) in the diet of guinea pigs shows a probiotic effect with positive results on the health of the animals (Table 5 and Table 6), especially in the reduction in diarrhea and prevention of recurrent infections by pathogens in young animals [12,31,36]. Similar work in guinea pigs treated with multiple strains and rabbits treated with antibiotics and probiotics showed no difference in the reduction in pathogens in the digestive tract compared to control groups [37].

The pH values in the present study were lower in animals treated with microbial bioactive compounds, which may have had a positive effect on the digestive process and nutrient absorption in the gastrointestinal tract [39]. According to the literature, the maintenance of stable pH values is known to positively influence cellular homeostasis as well as nutrient absorption [5,11,30].

Conversely, alterations in luminal pH in the intestinal tract favor the development of pathogenic diseases [35]; thus, an elevated pH in the stomach and in the pyloric region of the duodenum favors the development of infections [38]. It has been shown that the pH of the small intestinal surface microclimate is the result of a dynamic balance between H^+^ secretion and absorption across the luminal membrane (under normal conditions, H^+^ secretion is considered to predominate over absorption) and the diffusion of mucosal fluids into the intestinal lumen [30,41].

### 4.3. Modification of the Microbiota and the Environment of the Digestive Tract

In the present study, it was possible to observe very marked changes in the gut microbiota (Table 6 and Table 7). Similar results were also reported by other studies, where a greater increase in probiotic bacteria was observed in the digestive tract of the birds when they consumed probiotic microorganisms [42]. In the present study, it was also observed that animals consuming microbial bioactive compounds reduced the presence of pathogenic microorganisms; at the same time, the amount of microorganisms (bacteria and yeasts) of the species introduced with the diet increased (Table 6), which probably promoted a greater growth of beneficial microflora in the digestive tract of the guinea pigs studied.

Probiotic microorganisms have the ability to attach to and grow in the intestinal epithelium [11,35,40]. This scenario could be observed in the present study in guinea pigs that received probiotics as a substrate for microbial additives in their diet, where in samples from the intestinal scraping cultured on MRS and M17 Agar, Nutrient Agar and Sabouraud Agar, higher numbers of administered microorganisms (*Lactobacillus*, *Sacharromyces* and *Kluyeveromyces*) grew with the diet, and decreases in the population of *E. coli* and other enterobacteria were not demonstrated in the study (Table 7). A reduction in pathogenic microorganisms decreases the risk of infection in animals consuming probiotics in their diet [1,8]. A reduction in the number of *E. coli* and *Salmonella* sp. strains [27] was reported when analyzing feces from piglets consuming *Lactobacillus* spp. and *B. subtillis* in their diets [5,7,30,42].

There is also confirmatory evidence of changes in the population of Gram-negative and Gram-positive bacteria as well as lactic acid bacteria (*Lactobacillus* spp.), spore-forming bacteria (*Bacillus* spp.) and yeasts in the digestive tract with the inclusion of commonly used probiotics [9,12,31]. Others demonstrate a reduction in the presence of pathogens such as *Staphylococcus*, *Enterococcus*, *Listeria* and *Salmonella* in the small intestine of animals consuming probiotics obtained from the combination of *Bifidobacterium*, *Bacillus* and *Lactobacillus* strains; an effect also observed in the present study (Table 6 and Table 7) [11,35,41].

The increased presence of microorganisms introduced with the animals’ diet could be related to the ability of probiotics to colonize different segments of the digestive tract organs observed in this study (Table 6 and Table 7), as one of the mechanisms of action of probiotic bacteria is the ability to colonize and change the environmental conditions of the digestive tract and, in response, reduce the survival of pathogenic bacteria in the host [37,38].

In this regard, other studies report the ability to coat receptor binding sites, thus preventing the intestinal epithelium from binding to pathogenic microorganisms [41]. In research with broiler chickens, the inclusion of *Lactobacillus* spp. increased the presence of these lactic acid bacteria in the digestive tract [26,35], results that could also be observed in the present study, where probiotic microorganisms had an increased presence in the different segments of the gastrointestinal tract of guinea pigs consuming microbial bioactive compounds [6,18], since the main mechanism of action of probiotic microorganisms is to reinforce the integrity of the intestinal barrier and prevent the development of pathogenic bacterial populations in the intestinal epithelium [11,42].

## 5. Conclusions

The inclusion of agroindustrial substrates fermented with lactic bacteria and yeasts (T1, T2 and T3) helped to obtain a higher weight gain of male guinea pigs in the growth and fattening stage. They also helped to significantly reduce the occurrence of diarrhea and deaths caused by pathogens, mainly enterobacteria. They reduced macroscopic lesions in the organs of the digestive tract and increased the weight in the small intestine (without cecal contents), liver, lungs and kidneys and improve their appearance. Changes were also observed in the microbiota of the stomach, small intestine, cecum and colon, where the presence of enterobacteria was reduced and microorganisms (*Lactobacillus* spp., *Saccharomyces* spp. and *Kluyveromyces* spp.) that were introduced with the feed were increased.

These results form the basis for the use of agroindustrial substrates fermented with lactic acid bacteria and yeasts in commercial guinea pig production farms.

Similar studies with other animal species at different production stages are recommended to verify the probiotic effect with the use of agroindustrial substrates fermented with lactic acid bacteria and yeasts. It is also important to carry out molecular studies (PCR) to verify the quantity of microorganisms present and the possibility of modifying the natural microbiota in the different animal species (*L. acidophilus*, *L. bulgaricus*, *S. cerevisaie* and *K. fragilis*) fed with a mixture of probiotic microorganisms. 

## Figures and Tables

**Table 1:** Treatments used in the study and their formulation.

Treatment	Codification	Variants
Control	T0	Substrate-free control of fermented agroindustrial waste (lactic acid bacteria and/or yeasts)
Bioadditive 1	T1	1.00 mL fermented agroindustrial waste substrate with *L. acidophilus* and *L. bulgaricus*
Bioadditive 2	T2	1.00 mL fermented agroindustrial waste substrate with *S. cerevisiae* and *K. fragilis*
Bioadditive 3	T3	1.00 mL fermented agroindustrial waste substrate with *L. acidophilus*, *L. bulgaricus*, *S. cerevisiae* and *K. fragilis*

**Table 2:** Amount of feed at each stage of production and bromatological composition of the diet used for guinea pigs.

Production Stage	A Quantity of Food Offered, g/Animal/Day	Nutritional Composition ^#^, %				
		CP	EE	CF	Ash	DM
I	100	19	5.88	12.5	5.85	87.82
II	150	17	5.84	12.8	5.67	88.52
III	200	16	5.85	12.6	5.68	87.64

CP, crude protein. EE, ether extract. CF, crude fiber. Ash. DM, dry matter. ^#^, Proximate chemical analysis was performed according to the methodology described by AOAC [21].

**Table 3:** Incidence of diarrhea and mortality in guinea pigs, evaluated at 30, 60 and 90 days of study, when fed a diet with agroindustrial waste substrate fermented with bacteria and yeasts.

Period Evaluated, d	Indicator, %	Treatments				SEM	*p*-Value
		T0	T1	T2	T3		
30	Incidence of diarrhea	4.08 ^a^	1.08 ^c^	0.9 ^c^	0.3 ^b^	2.2	0.015
	Mortality	0.17	0.58	-	-	-	-
60	Incidence of diarrhea	2.62 ^b^	1.40 ^c^	0.72 ^a^	0.19 ^b^	2.5	0.014
	Mortality	0.58	0.52	-	-	-	-
90	Incidence of diarrhea	1.03	-	-	-	-	-
	Mortality	-	-	-	-	-	-

^a,b,c^ superscripts different in the same column differ at *p* < 0.05 [34]. T0, control. T1, *L. acidophilus* and *L. bulgaricus*. T2, *S. cerevisiae* and *K. fragilis*. T3, *L. acidophilus*, *L. bulgariccus*, *S. cerevisiae* and *K. fragilis*. d, day.

**Table 4:** Relative organ weights of the digestive tract of 120-day-old guinea pigs fed a diet containing agroindustrial waste substrate fermented with bacteria and yeast.

Organs of the Digestive Tract, g	Treatments (n = 6 per Treatments)				SEM	*p*-Value
	T0	T1	T2	T3		
Stomach with luminal contents	52.06	52.23	54.12	54.51	2.11	0.084
Stomach without luminal contents	8.15	8.24	8.73	8.97	1.28	0.059
Small intestine with luminal contents	40.21 ^b^	43.32 ^a^	42.43 ^ab^	43.42 ^a^	0.18	0.012
Small intestine without luminal contents	25.64	24.76	25.87	26.32	0.12	0.081
Large intestine with luminal contents	54.12	55.02	54.87	55.15	0.25	0.073
Large intestine without luminal contents	22.09	21.98	23.45	22.97	0.10	0.342
Cecum with luminal contents	85.13	86.02	86.03	86.05	0.15	0.061
Cecum without luminal contents	22.86	23.07	23.26	23.44	0.21	0.842
Liver	34.02 ^c^	34.56 ^c^	34.98 ^b^	35.23 ^a^	0.54	0.012
Lungs	13.87 ^b^	14.24 ^b^	14.87 ^a^	15.34 ^a^	0.22	0.008
Kidneys	10.02 ^c^	10.24 ^c^	10.67 ^b^	11.64 ^a^	0.10	0.022

^a,b,c^ superscripts different in the same column differ at *p* < 0.05 [34]. T0, control. T1, *L. acidophilus* and *L. bulgaricus*. T2, *S. cerevisiae* and *K. fragilis*. T3, *L. acidophilus*, *L. bulgariccus*, *S. cerevisiae* and *K. fragilis*.

**Table 5:** Macroscopic lesions observed in 120-day-old guinea pigs (n = 6 per treatments) fed a diet containing agroindustrial waste substrate fermented with bacteria and yeasts.

Parameter	Stomach				Small Intestine				Colon				Cecum			
	T0	T1	T2	T3	T0	T1	T2	T3	T0	T1	T2	T3	T0	T1	T2	T3
Gut wall thickness																
Altered	1.08	WI	WI	WI	0.87	WI	WI	WI	1.06	WI	WI	WI	2.08	WI	WI	WI
Circulatory disorders of the mucosa																
Edema	1.02	WI	WI	WI	1.21	WI	WI	WI	0.28	WI	WI	WI	0.24	WI	WI	WI
Congestion	0.15	WI	WI	WI	0.89	WI	WI	WI	0.06	WI	WI	WI	0.36	WI	WI	WI
Hemorrhage	0.08	WI	WI	WI	0.31	WI	WI	WI	WI	WI	WI	WI	0.18	WI	WI	WI
Intestinal contents																
Aqueous	0.15	WI	WI	WI	0.09	WI	WI	WI	0.09	WI	WI	WI	0.31	WI	WI	WI
Mucous	0.10	WI	WI	WI	0.24	WI	WI	WI	0.18	WI	WI	WI	0.25	WI	WI	WI
Frothy	0.18	WI	WI	WI	0.23	WI	WI	WI	0.37	WI	WI	WI	0.33	WI	WI	WI
pH	2.9	1.8	1.7	1.8	5.5	4.8	4.7	4.8	6.18	5.67	5.65	5.71	6.3	5.68	5.67	5.68

T0, control. T1, *L. acidophilus* and *L. bulgaricus*. T2, *S. cerevisiae* and *K. fragilis*. T3, *L. acidophilus*, *L. bulgariccus*, *S. cerevisiae* and *K. fragilis*. When there were no significant macroscopic lesions in the organs of the gastrointestinal tract, the following abbreviation WI (no lesions) was used. WI, without injury.

**Table 6:** Microbial load in each segment of the digestive tract, cultured in Petri dishes with culture media (MRS, M17, Nutrient, Sabouraud Dextrose and MacConkey Agar, respectively) of samples from stomach, intestine, colon and cecum scrapings from 120-day-old guinea pigs (*post-mortem*).

Organs of the Digestive System	Culture Medium for Microbial Growth	Treatments, log^−1^				SEM	*p*-Value
		T0	T1	T2	T3		
Stomach	*Lactobacillus* spp.	6.22	6.31	6.32	6.42	0.32	0.841
	*Lactobacillus* spp.	6.47	6.48	6.50	6.72	0.55	0.836
	*kluyveromyces* spp.	6.90	6.87	6.85	6.77	0.28	0.965
	*Saccharomyces* spp.	2.80	2.65	2.57	3.50	0.26	0.077
	*Enterobacteriaceae*	5.97 ^a^	5.59 ^a^	4.47 ^b^	3.45 ^b^	0.29	0.003
Small intestine	*Lactobacillus* spp.	3.77 ^b^	6.51 ^a^	6.62 ^a^	7.07 ^a^	0.46	<0.001
	*Lactobacillus* spp.	3.72 ^b^	6.97 ^a^	7.07 ^a^	7.15 ^a^	0.14	<0.001
	*kluyveromyces* spp.	6.72	6.58	6.72	6.70	0.27	0.081
	*Saccharomyces* spp.	3.05 ^b^	5.98 ^a^	6.10 ^a^	6.15 ^a^	0.26	<0.001
	*Enterobacteriaceae*	6.20 ^a^	3.08 ^b^	3.42^b^	2.85 ^b^	0.23	<0.001
Colon	*Lactobacillus* spp.	3.40 ^b^	7.08 ^a^	6.95 ^a^	7.12 ^a^	0.37	<0.001
	*Lactobacillus* spp.	4.92 ^b^	7.02 ^a^	7.05 ^a^	6.97b ^a^	1.15	0.030
	*kluyveromyces* spp.	6.85	6.66	6.85	6.85	0.61	0.510
	*Saccharomyces* spp.	2.27 ^b^	5.98 ^a^	5.57 ^a^	5.85 ^a^	0.12	<0.001
	*Enterobacteriaceae*	6.45 ^a^	4.15 ^b^	3.95 ^b^	3.98 ^b^	0.10	<0.001
Cecum	*Lactobacillus* spp.	4.07 ^b^	7.18 ^a^	7.07 ^a^	7.15 ^a^	0.10	<0.001
	*Lactobacillus* spp.	3.47 ^b^	6.97 ^a^	7.15 ^a^	6.95 ^a^	0.23	0.002
	*kluyveromyces* spp.	6.37	6.52	6.12	6.97	0.20	0.505
	*Saccharomyces* spp.	2.37 ^c^	5.83 ^a^	5.61 ^b^	5.97 ^a^	0.15	<0.001
	*Enterobacteriaceae*	6.87 ^a^	3.43 ^b^	3.30 ^b^	3.52 ^b^	0.24	0.007

^a,b,c^ superscripts different in the same column differ at *p* < 0.05 [34]. T0, control. T1, *L. acidophilus* and *L. bulgaricus*. T2, *S. cerevisiae* and *K. fragilis*. T3, *L. acidophilus*, *L. bulgariccus*, *S. cerevisiae* and *K. fragilis*. log^−1^, these values correspond to logarithmic scales.

**Table 7:** Main microorganisms detected in samples from the scraping of the digestive tract of 120-day-old guinea pigs and associated with bacteria and yeasts introduced with the diet, identified using the API (BioMerieux) biochemical identification system.

API	Numerical Profile	Microorganism	Stomach (n = 6 per Treatments)				Small Intestine (n = 6 per Treatments)				Cecum (n = 6 per Treatments)			
			T0	T1	T2	T3	T0	T1	T2	T3	T0	T1	T2	T3
API 50 CHL	4356101	*Lactobacillus* spp.	***	**	**	**	*	***	***	***	*	**	**	**
	3322230	*Lactobacillus* spp.	*	**	***	**	*	***	***	***	*	**	**	**
	1552137	*L. bulgariccus*		**	**	**	*	***	***	***	*	**	**	**
	1231576	*L. lactis*		**	**	**	*	*	*	*	*		*	*
	1269781	*P. acidilactici*	*	*	*	*	*	**	**	**	*	*		*
	4356135	*L. acidophilus*		*	*	*		**	**	**	*	*	*	*
	1184227	*L. bulgariccus*		**	**	**		***	***	***		*	*	*
	4356101	*S. thermophillus*		*	*	*		*	*	*		*		*
	2530294	*L. paracasei*	*		*		*	*	*	*	*			*
	5310336	*L. rhamnosus*	*			*	*	*	*	*	*		*	
	8042697	*Pediococcus* spp.	*			*	*			*	*	*		*
API 20 E	1427157	*E. coli*	***	*	*		***	*	*		**		*	*
	1431430	*E. coli*	**	*	*	*	***	*	*	*	*	*	*	*
	1429274	*E. coli*	*	*	*	*	**	*	*		*	*	*	*
	1177524	*E. coli*	**		*	*	**	*	*	*	*	*		
	4313717	*P. multicida*	*	*	*		*	*	*					
	1313837	*S. dysenteriae*	**	*		*	*			*	*	*		
API ID 32 C	5764734	*Saccharomyces* spp.	*	**	**	**	*	**	***	***		**	**	***
	4026727	*Saccharomyces* spp.	*	**	**	**	*	**	**	**	*	*	**	
	9763534	*S. cerevisiae*	*	**	**	***	*	**	***	***	*	*	*	**
	7401236	*S. boulardii*	*	*	*	*	*	*	**	**		*	**	
	2009655	*Kluyveromyces* spp.		*	**	***		*	***	***		*	***	***
	2229633	*K. marxianus*	*	*	*	*		*	***	***	*	*	**	**
	4653746	*K. fragilis*			**	***			***	***			***	***
	3659028	*Candida* spp.	*	*		*	*		*	*	*	*		

T0, control. T1, *L. acidophilus* and *L. bulgaricus*. T2, *S. cerevisiae* and *K. fragilis*. T3, *L. acidophilus*, *L. bulgariccus*, *S. cerevisiae* and *K. fragilis*. ***, excellent. **, very good. *, good.

## Data Availability

Data are contained within the article.
